# Endogenous Retroviruses in Fish Genomes: From Relics of Past Infections to Evolutionary Innovations?

**DOI:** 10.3389/fmicb.2016.01197

**Published:** 2016-08-09

**Authors:** Magali Naville, Jean-Nicolas Volff

**Affiliations:** Génomique Évolutive des Poissons, Institut de Génomique Fonctionnelle de Lyon, École Normale Supérieure de Lyon, CNRS, Université Lyon 1Lyon, France

**Keywords:** teleost fish, coelacanth, sharks, genome, evolution, retroviridae, retroviruses, endogenous retroviruses

## Abstract

The increasing availability of fish genome sequences has allowed to gain new insights into the diversity and host distribution of retroviruses in fish and other vertebrates. This distribution can be assessed through the identification and analysis of endogenous retroviruses, which are proviral remnants of past infections integrated in genomes. Retroviral sequences are probably important for evolution through their ability to induce rearrangements and to contribute regulatory and coding sequences; they may also protect their host against new infections. We argue that the current mass of genome sequences will soon strongly improve our understanding of retrovirus diversity and evolution in aquatic animals, with the identification of new/re-emerging elements and host resistance genes that restrict their infectivity.

## Introduction

Understanding the infection history of living organisms is essential to apprehend the (co)evolution of infection agents with their hosts, in particular on key aspects such as immunity. To this regard, viruses, which can spread between individuals and often cause disease, constitute a major point of interest.

Viruses form the most numerous and diverse group of genetic entities. They are generally constituted by single- or double-stranded RNA or DNA genomes embedded within a protein coat. Among them, retroviruses consist in single-stranded positive-sense RNA viruses with a DNA intermediate. After infection, a retrovirus reaches the cytoplasm of the host cell and produces DNA through the reverse transcription of its RNA genome into complementary DNA (cDNA). This step is catalyzed by the reverse transcriptase (RT), an RNA-dependent DNA polymerase that is generally encoded by the retrovirus itself. Subsequently, the cDNA molecule is integrated into the host nuclear genome through the action of the retroviral integrase, forming a provirus. Provirus genes are transcribed and translated by the host machinery, like classical cellular genes.

Retroviruses are delimited by long terminal repeats (LTRs), which carry a promoter sequence and are involved in interactions with the integrase for insertion. Retrovirus genomes classically contain three open reading frames: *gag* (5′, group-specific antigen), encoding core and structural proteins, *pol* (polymerase), producing a polyprotein with RT, protease and integrase domains, and *env* (3′, envelope), which codes for coat proteins. Additional accessory proteins are encoded by more complex retroviruses. After integration, ectopic homologous recombination can occur between both LTRs. This eliminates one LTR copy and the intervening sequence, generating the solo LTRs that are frequently found in genomes. Evolutionary switch can occur between retroviruses and non-infectious LTR retrotransposons through the gain or loss of envelope genes (Malik et al., 2000).

If they have infected the germ line, integrated proviruses, also called endogenous retroviruses (ERVs), can be maintained within host genomes over millions of years by “vertical” transmission from parents to offspring (Feschotte and Gilbert, 2012). Identifying ERVs in genomes, which is now facilitated by the ever increasing amount of sequence data, provides an idea of the infection history by retroviruses and might give some hints on their past and current diversity, as well as on their mutation rate (Patel et al., 2011; Aiewsakun and Katzourakis, 2015). This can also help to reassess the age of retrovirus groups and to understand the coevolution with their hosts (Katzourakis et al., 2009; Keckesova et al., 2009; Gilbert and Feschotte, 2010). Finally, such genomic analyses might identify so far unknown retroviruses and full-length elements with the potential for retained infectivity and reemergence (Wildschutte et al., 2016). The analysis of relics of past infections in genomes, also called “viral fossil record”, has been called paleovirology (Patel et al., 2011).

## Vertebrate Endogenous Retroviruses

**FIGURE 1:**
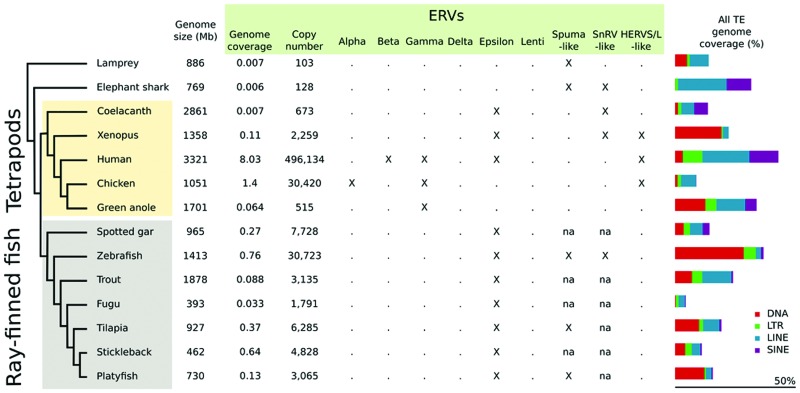
**Endogenous retrovirus (ERV) genome coverage, copy number, and family distribution in different vertebrate species.** Values were retrieved from Chalopin et al. (2015). na: not available. The distinction between SnRV-like and Spuma-like elements requires further investigation, since the SnRV-like clade has been defined only recently (Hayward et al., 2015).

Endogenous retroviruses have been identified in all vertebrate lineages (Hayward et al., 2015). Despite the relatively low frequency of integration that is expected in germ cells compared to somatic cells, ERVs are major components of some tetrapod genomes, constituting with over 100.000 integrated elements as much as 8% of the human genome (**Figure 1**). ERVs are also present in birds, reptiles and amphibians, with higher genome content in birds: with 30,420 elements, they cover 1.4% of the chicken genome, compared to 0.1% of the *Xenopus* genome (ca. 2,300 elements; Holmes, 2011; Chalopin et al., 2015) (**Figure 1**).

**FIGURE 2:**
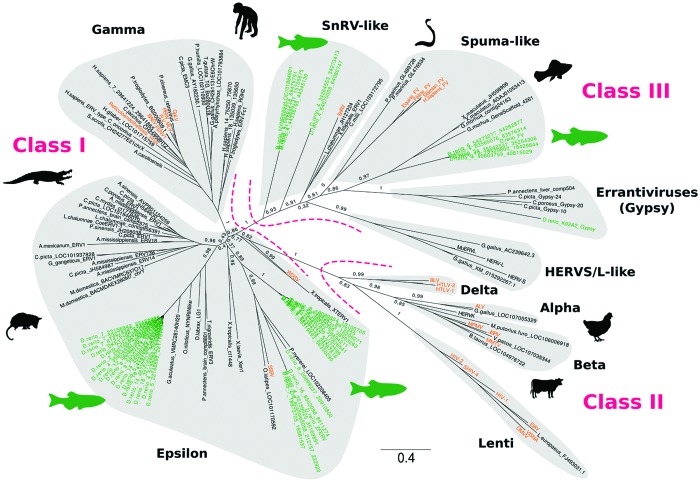
**Phylogenetic analysis of zebrafish ERVs and other fish retroelements.** The phylogeny was reconstructed from an alignment of RT (201 amino acids, translated from element copies) by Maximum Likelihood using PhyML (Guindon et al., 2010) with optimized parameters (best of NNI and SPR; optimized invariable sites). Branch values represent supporting aLRT non-parametric statistics. Zebrafish and exogenous retrovirus sequences are highlighted in green and orange, respectively. Gypsy LTR retrotransposon sequences were used as an outgroup. The tree was drawn using FigTree (http://tree.bio.ed.ac.uk/software/figtree/).

In vertebrates, all retroviruses belong to a single large family called Retroviridae, possibly of monophyletic origin. Retroviridae are subdivided into two subfamilies (Orthoretrovirinae and Spumaretrovirinae) and seven genera: Alpharetroviruses, Betaretroviruses, Gammaretroviruses, Deltaretroviruses, Epsilonretroviruses, Lentiviruses, and Spumaviruses (**Figure 2**). While some vertebrate ERVs are too divergent to be assigned to specific exogenous retrovirus genera, ERVs have been classified into three main different classes based on phylogenetic evidence: class I, with elements related to Gamma- and Epsilonretroviruses, class II, including elements grouping with Lentiviruses, Alpha-, Beta-, and Deltaretroviruses, and class III, with *Spumavirus*-like sequences, which represents the most divergent retroviral group (Gifford et al., 2005; Hayward et al., 2015) (**Figure 2**). Two additional clades have been recently recognized: HERVS/L (Human endogenous retroviruses S/L)-like and SnRV (Snakehead fish retrovirus)-like elements (Hayward et al., 2015).

Lineage-specific differences in ERV clade distribution have been observed in tetrapods (Herniou et al., 1998; Gifford et al., 2005; Katzourakis et al., 2009; Hayward et al., 2015). Class I elements are widespread in tetrapods, with a stronger contribution of Gammaretroviruses compared to Epsilonretroviruses. Class II ERVs are largely confined to mammals and birds, with high copy number of Betaretroviruses in mammals and Alpharetroviruses in birds and alligator (**Figure 2**). Lentivirus-like ERVs are found in lagomorphs and carnivores, while Delta-like ERV sequences have not been reported so far (Hayward et al., 2015). Insertions of Foamy virus (*Spumavirus*) have been identified in the genomes of some mammals (Katzourakis et al., 2009; Han and Worobey, 2012b). HERVS/L-like sequences are present in almost all tetrapods tested, and SnRV-like elements are found in some reptiles (Hayward et al., 2015) (**Figures 1** and **2**).

The Retroviridae family is restricted to vertebrates. However, other types of ERVs of independent origins have been detected in tunicates, which are the closest living relatives of vertebrates within chordates. Two divergent ERV families have been identified in the genome of the marine appendicularian *Oikopleura dioica*, one of them having possibly gained its envelope gene from a paramyxovirus (RNA virus; Volff et al., 2004; Henriet et al., 2015). Retroviruses of independent origins have been detected in more divergent invertebrates and in plants (Malik et al., 2000).

## Fish Endogenous Retroviruses

Fish are, like other vertebrates, infected by exogenous retroviruses. Retroviruses have been found to be associated with tumors in fish intensively cultured for food, in wild fish populations showing signs of sickness, and in fish reared in the laboratory (Lepa and Siwicki, 2011; Coffee et al., 2013). One example is the Walleye dermal sarcoma virus (WDSV), an *Epsilonretrovirus* associated with skin tumors in the walleye, a freshwater perciform (Walker, 1969). The snakehead retrovirus (SnRV) has been identified in a cell line derived from the striped snakehead fish (Frerichs et al., 1991). The SSSV virus has been isolated from Atlantic salmon swim bladder sarcomas (Paul et al., 2006).

The genomes of different fish species have been analyzed for the presence of ERV sequences (Chalopin et al., 2015). The species with sequenced genomes analyzed included (from the most related to human to the most divergent): the coelacanth, which is a lobe-finned fish related to tetrapods; teleost fish and other ray-finned fish; cartilaginous fish including sharks; and the sea lamprey, which is a jawless fish.

Altogether, ERV sequences are present in fish genomes, but with a lower content than in mammals (1-0.01% of the genome depending on the species; Chalopin et al., 2015) (**Figure 1**). In teleosts ERV contribution to genomes typically ranges from 0.033% in the compact Fugu genome, with ca. 1,800 insertions, to 0.76% in zebrafish, with more than 30,000 insertions. ERV content is an order of magnitude lower in elephant shark, coelacanth and lamprey, with a genome coverage of ca. 0.007% and 100–700 insertions. Epsilon-like (class I) and *Spumavirus*-related (class III) ERVs are the major retroviral elements found in fish genomes (**Figure 2**). A more divergent ERV clade was found in the genomes of zebrafish, coelacanth, sea lamprey but also amphibians, which might represent an ancestral branch of vertebrate ERVs and encompasses SnRV retroviruses (Hayward et al., 2015).

### Coelacanth

The major category of ERVs in coelacanth is constituted by SnRV-like retroviruses, but Epsilon-like elements are also present (Hayward et al., 2015). One insertion of an Epsilon-like element called CoeERV1-1 has been found at orthologous positions in the genomes of the two extant species of coelacanths, suggesting an at least 6–8 million year-old integration (Naville et al., 2014). Complete CoeERV1-1 elements and LTRs are 7.2 kb and 475 nt in length, respectively. 258 fragments of variable sizes similar to CoeERV1-1 have been identified in the whole genome of the African coelacanth *Latimeria chalumnae*, most of them being internally deleted or present as solo LTRs. Interestingly, CoeERV1-1 sequences are closely related to turtle and crocodile ERV sequences (**Figure 2**). This suggests horizontal transfer between reptiles and coelacanths, or infection of both lineages by related retroviruses (Naville et al., 2014).

In addition, an endogenous foamy virus-like element (*Spumavirus*) called CoeEFV has been identified in the genome of the African coelacanth (Han and Worobey, 2012a). Beside *gag*, *pol*, and *env*, two additional putative open reading frames have been detected at positions similar to mammalian foamy virus accessory genes but with no significant similarity. CoeEFV probably invaded the coelacanth genome more than 19 million years ago (Han and Worobey, 2012a).

### Ray-Finned Fish

Ray-finned fish genomes mostly contain Epsilon-like sequences, with different sublineages (Herniou et al., 1998; Basta et al., 2009; **Figures 1** and **2**). Some of these sequences occasionally include additional open reading frames, encoding for example a 2′,3′-cyclic nucleotide 3′-phosphodiesterase (CNPase) or macro domain proteins (Basta et al., 2009). ERVs have been found in different ray-finned fish species, including an element showing 200–300 copies in the very compact genome of the pufferfish *Tetraodon nigroviridis* (Fischer et al., 2005).

The best studied Epsilon-line ERV in teleost fish is ZFERV from zebrafish (Shen and Steiner, 2004) (**Figure 2**). The ZFERV provirus is 11.2 kb in length and phylogenetically related to the salmon swim bladder sarcoma virus (SSSV). Transcription is predominantly detected in the thymus of both larval and adult fish, under the form of several transcripts. ZFERV is amplified in zebrafish T-cell leukemia (Frazer et al., 2012). The fusion core of the ZFERV envelope protein has been characterized at the functional level (Shi et al., 2015). In addition to ZFERV-related elements, we could detect two additional families of Epsilon-like sequences in the zebrafish genome that clearly segregate in a phylogeny, indicating the presence of divergent ERVs in this fish (**Figure 2**). The high degree of similarity between ERV elements within each phylogenetic group suggests recent introduction and/or spreading in the zebrafish genome.

Endogenous Foamy virus (EFV) sequences (Spumaviruses) have been detected in the genome of several teleost fish species including the platyfish, the zebrafish and the Atlantic cod (Llorens et al., 2009; Schartl et al., 2013) (**Figure 2**). The molecular phylogeny of EFVs is consistent with the host phylogeny; their distribution supports an ancient marine evolutionary origin, with possible host-virus coevolution. In the platyfish *Xiphophorus maculatus*, several almost intact envelope-encoding copies are present (Schartl et al., 2013). The presence of nearly non-corrupted elements in divergent teleost species and the patchy distribution of the virus suggest independent infectious introductions into the germ-line. Even if exogenous Foamy viruses have not been described so far for teleosts, exogenous versions of the virus might have been recently active and could still be infectious in ray-finned fishes.

Finally, the zebrafish genome contains several SnRV-like sequences, possibly with two distinct families (**Figures 1** and **2**). Neither Gamma sequences nor class II elements have been detected so far in teleosts (Hayward et al., 2015).

### Cartilaginous Fish

Screening of the genome of the elephant shark *Callorhinchus milii* for retroviral sequences (CmiERVs) has revealed the presence of three (nearly) complete ERV insertions and many short ERV fragments (Han, 2015). Phylogenetic analysis revealed three major lineages, one clustering with the snakehead fish retrovirus SnRV (**Figure 2**), and two grouping with Epsilonretroviruses from walleye and amphibians (Han, 2015).

### Lamprey

Very divergent elements were found in the genome of the lamprey; they group together with Spumaviruses according to our RT-based phylogeny, but with only a very low support (**Figure 2**). ERV abundance is lower in the lamprey compared to most other vertebrates (Hayward et al., 2015) (**Figure 1**).

## Do Fish ERVs Fulfil Functions Useful for Their Host?

While most ERVs have no clear role for their hosts, recent results have demonstrated beneficial functions having increased the fitness of the organism (Warren et al., 2015; Naville et al., 2016). Such data have been obtained in mammals, but almost nothing is known on potentially useful roles on genome function and evolution in fish. Given the common evolutionary origin of mammals and fish ERVs, we believe that these sequences could have advantageous functions in fish too.

As mobile and repeated sequences, ERVs are mediators of genomic plasticity (Goodier and Kazazian, 2008). They can disrupt sequences through insertion, and can recombine to mediate DNA rearrangements. Such events might destroy important genomic sequences and negatively affect the fitness of their hosts. On the other hand, the effect of ERVs on genome plasticity might catalyze genome evolution and generate advantageous rearrangements, for example new duplicated genes or gene combinations, with a possible role in speciation. In primates, ERVs have mediated genomic rearrangements during evolution (Hughes and Coffin, 2001). Interestingly, homologous recombination between LTRs has been shown to be involved in post-speciation genome divergence in coelacanths (Naville et al., 2014).

In mammals, ERVs and other transposable elements are able to modify gene expression through the regulatory sequences they carry. They can even put multiple genes under a same new regulation and rewire complete regulatory networks (Feschotte, 2008). For instance, lineage-specific ERVs have dispersed multiple interferon-inducible enhancers independently in different mammalian genomes (Chuong et al., 2016). Again, nothing is known about such a role for ERVs in fish. However, another transposable element, a non-autonomous DNA transposon called EnSpmN6_DR, has shaped the repertoire of p53 target genes in zebrafish through the spreading of embedded p53-responsive elements to the vicinity of genes (Micale et al., 2012). This indicates that regulatory network rewiring by transposable elements, and potentially by ERVs, is possible in fish.

Endogenous retroviruses (ERVs) and other transposable elements have been described as a source of non-coding RNA genes, including microRNA (miRNA) and long non-coding RNA (lncRNA) genes. A strong contribution of ERVs to non-coding RNA genes has been observed in human and mouse stem cells (Fort et al., 2014). Only 5% of miRNA are derived from transposable elements in fish (compared to ca. 20% in human), mostly from DNA transposons (Qin et al., 2015). About 20% of zebrafish lncRNAs contain TE sequences, but with little contribution of LTR elements (Kapusta et al., 2013). The reduced contribution of ERV-like sequences is not completely surprising, since the zebrafish genome is dominated by DNA transposons (Chalopin et al., 2015).

Finally, retroviruses and other transposable elements have been shown to serve as new protein-coding genes during evolution in vertebrates and other lineages. Some of these genes have important functions for their host (Volff, 2006; Aswad and Katzourakis, 2012; Warren et al., 2015; Naville et al., 2016). In mammals, genes derived from endogenous retrovirus envelope sequences encode proteins called Syncytins that are involved in the formation of the placenta (Lavialle et al., 2013). Almost nothing is known on retrovirus-derived genes in fish. However, a gene called *Gin2*, derived from an integrase, has been identified in fish and some other vertebrate lineages (Marín, 2010; Chalopin et al., 2012). This gene was detected in all ray-finned fish species tested as well as in coelacanth and cartilaginous fish (elephant shark; Chalopin et al., 2012). The HHCC zinc finger from the ancestral integrase has been kept, suggesting that the GIN2 protein is able to bind DNA or RNA. *Gin2* is expressed during gastrulation in zebrafish (Chalopin et al., 2012).

Interestingly, some retrovirus-derived genes are involved in resistance against retroviral infections in vertebrates. This is the case for the mouse Friend virus susceptibility-1 (*Fv1*) gene (Best et al., 1996). *Fv1*, which is derived from the *gag* gene of the MERV-L endogenous retrovirus family, protects the host against infection by the murine leukaemia virus (MLV) and other types of retroviruses. The Fv1 protein blocks MLV infection through interaction with the capsid protein (Nair and Rein, 2014). Other genes derived from ERV *gag* and *env* genes are involved in resistance against retroviral infection in mouse, sheep, cat, and chicken (Varela et al., 2009; Aswad and Katzourakis, 2012). We propose that retrovirus-derived genes that are still to be identified might be involved in resistance against retroviral infections in fish too.

## Conclusion

This survey of ERVs highlights the relative paucity of knowledge in fish compared to mammals, and a number of missing data. In particular, several ERV groups lack any related exogenous viruses from which they would have originated. As an example, no fish Foamy virus has been described, despite the presence of endogenous foamy viruses in several fish species. This illustrates well how useful is the analysis of endogenous viral sequences in genomes to better characterize the diversity of infectious agents. Further studies on new “aquatic” genomes may uncover additional families or even new types of viruses. Comparative analysis of new genomes will also help reevaluating the age and evolutionary history of retroviruses. For example, detection of Foamy-like endogenous elements in fish strongly supports an ancient marine evolutionary origin (Schartl et al., 2013).

Much work is also required to better understand the evolutionary impact of ERVs on fish genomes: how they contribute to the evolution of genome architecture, RNA and protein repertoire and regulatory networks. Strong effects have been reported in mammals, which present a much lower level of diversity of transposable elements than fish (Chalopin et al., 2015). ERVs and other types of mobile and repeated sequences have the potential to play a very significant role in the huge level of biological diversity observed in fish, which affects many aspects including development, morphology, physiology, behavior, reproduction, and ecology (Volff, 2005). Of particular interest for the aquaculture are retrovirus-derived resistance genes restricting new infections (Aswad and Katzourakis, 2012; Chuong et al., 2016). Candidates for such genes could be identified through a screening of genomes for virus-derived sequences that can then be tested at the functional level.

Finally, recent work suggests that a similar analysis can be also applied to RNA viruses without DNA stage and to DNA viruses, for which genomic integrated forms have been also detected in vertebrates (Katzourakis and Gifford, 2010; Holmes, 2011). Accordingly, DNA sequences related to Parvoviruses, which possess linear single-stranded DNA genomes, have been identified in the genome of a pufferfish (Liu et al., 2011). The functional interconnections between host and viruses within genomes in fish might thus be of wider significance than previously thought.

## Author Contributions

J-NV and MN have drafted the manuscript, MN has analyzed sequence data.

## Conflict of Interest Statement

The authors declare that the research was conducted in the absence of any commercial or financial relationships that could be construed as a potential conflict of interest.

## References

[B1] Aiewsakun P. Katzourakis A. (2015). Endogenous viruses: Connecting recent and ancient viral evolution. *Virology* 479–480, 26–37. 10.1016/j.virol.2015.02.011 25771486

[B2] Aswad A. Katzourakis A. (2012). Paleovirology and virally derived immunity. *Trends Ecol. Evol.* 27 627–636. 10.1016/j.tree.2012.07.007 22901901

[B3] Basta H. A. Cleveland S. B. Clinton R. A. Dimitrov A. G. McClure M. A. (2009). Evolution of teleost fish retroviruses: characterization of new retroviruses with cellular genes. *J. Virol.* 83 10152–10162. 10.1128/JVI.02546-08 19625413 PMC2748043

[B4] Best S. Tissier P. L. Towers G. Stoye J. P. (1996). Positional cloning of the mouse retrovirus restriction gene Fvl. *Nature* 382 826–829. 10.1038/382826a0 8752279

[B5] Chalopin D. Galiana D. Volff J.-N. (2012). Genetic innovation in vertebrates: gypsy integrase genes and other genes derived from transposable elements. *Int. J. Evol. Biol.* 2012:724519 10.1155/2012/724519 PMC3424704 22928150

[B6] Chalopin D. Naville M. Plard F. Galiana D. Volff J. N. (2015). Comparative analysis of transposable elements highlights mobilome diversity and evolution in vertebrates. *Genome Biol. Evol.* 7 567–580. 10.1093/gbe/evv005 25577199 PMC4350176

[B7] Chuong E. B. Elde N. C. Feschotte C. (2016). Regulatory evolution of innate immunity through co-option of endogenous retroviruses. *Science* 351 1083–1087. 10.1126/science.aad5497 26941318 PMC4887275

[B8] Coffee L. L. Casey J. W. Bowser P. R. (2013). Pathology of tumors in fish associated with retroviruses: a review. *Vet. Pathol.* 50 390–403. 10.1177/0300985813480529 23456970

[B9] Feschotte C. (2008). Transposable elements and the evolution of regulatory networks. *Nat. Rev. Genet.* 9 397–405. 10.1038/nrg2337 18368054 PMC2596197

[B10] Feschotte C. Gilbert C. (2012). Endogenous viruses: insights into viral evolution and impact on host biology. *Nat. Rev. Genet.* 13 283–296. 10.1038/nrg3199 22421730

[B11] Fischer C. Bouneau L. Coutanceau J.-P. Weissenbach J. Ozouf-Costaz C. Volff J.-N. (2005). Diversity and clustered distribution of retrotransposable elements in the compact genome of the pufferfish *Tetraodon nigroviridis*. *Cytogenet. Genome Res.* 110 522–536. 10.1159/000084985 16093705

[B12] Fort A. Hashimoto K. Yamada D. Salimullah M. Keya C. A. Saxena A. (2014). Deep transcriptome profiling of mammalian stem cells supports a regulatory role for retrotransposons in pluripotency maintenance. *Nat. Genet.* 46 558–566. 10.1038/ng.2965 24777452

[B13] Frazer J. K. Batchelor L. A. Bradley D. F. Brown K. H. Dobrinski K. P. Lee C. (2012). Genomic amplification of an endogenous retrovirus in zebrafish t-cell malignancies, Genomic Amplification of an Endogenous Retrovirus in Zebrafish T-Cell Malignancies. *Adv. Hematol.* 2012:e627920 10.1155/2012/627920 PMC3382231 22745640

[B14] Frerichs G. N. Morgan D. Hart D. Skerrow C. Roberts R. J. Onions D. E. (1991). Spontaneously productive C-type retrovirus infection of fish cell lines. *J. Gen. Virol.* 72 (Pt 10), 2537–2539. 10.1099/0022-1317-72-10-2537 1717644

[B15] Gifford R. Kabat P. Martin J. Lynch C. Tristem M. (2005). Evolution and distribution of class II-related endogenous retroviruses. *J. Virol.* 79 6478–6486. 10.1128/JVI.79.10.6478-6486.2005 15858031 PMC1091674

[B16] Gilbert C. Feschotte C. (2010). Genomic fossils calibrate the long-term evolution of hepadnaviruses. *PLoS Biol.* 8:e1000495 10.1371/journal.pbio.1000495 PMC2946954 20927357

[B17] Goodier J. L. Kazazian H. H. Jr. (2008). Retrotransposons revisited: the restraint and rehabilitation of parasites. *Cell* 135 23–35. 10.1016/j.cell.2008.09.022 18854152

[B18] Guindon S. Dufayard J.-F. Lefort V. Anisimova M. Hordijk W. Gascuel O. (2010). New algorithms and methods to estimate maximum-likelihood phylogenies: assessing the performance of phyml 3.0. *Syst. Biol.* 59 307–321. 10.1093/sysbio/syq010 20525638

[B19] Han G.-Z. (2015). Extensive retroviral diversity in shark. *Retrovirology* 12:34 10.1186/s12977-015-0158-4 PMC4422223 25927737

[B20] Han G.-Z. Worobey M. (2012a). An endogenous foamy-like viral element in the coelacanth genome. *PLoS Pathog.* 8:e1002790 10.1371/journal.ppat.1002790 PMC3386198 22761578

[B21] Han G.-Z. Worobey M. (2012b). An endogenous foamy virus in the aye-aye (*Daubentonia madagascariensis*). *J. Virol.* 86 7696–7698. 10.1128/JVI.00650-12 22573860 PMC3416287

[B22] Hayward A. Cornwallis C. K. Jern P. (2015). Pan-vertebrate comparative genomics unmasks retrovirus macroevolution. *Proc. Natl. Acad. Sci. U.S.A.* 112 464–469. 10.1073/pnas.1414980112 25535393 PMC4299219

[B23] Henriet S. Sumic S. Doufoundou-Guilengui C. Jensen M. F. Grandmougin C. Fal K. (2015). Embryonic expression of endogenous retroviral RNAs in somatic tissues adjacent to the *Oikopleura* germline. *Nucleic Acids Res.* 43 3701–3711. 10.1093/nar/gkv169 25779047 PMC4402516

[B24] Herniou E. Martin J. Miller K. Cook J. Wilkinson M. Tristem M. (1998). Retroviral diversity and distribution in vertebrates. *J. Virol.* 72 5955–5966. 10.1128/jvi.72.7.5955-5966.1998 PMC110400 9621058

[B25] Holmes E. C. (2011). The evolution of endogenous viral elements. *Cell Host Microbe* 10 368–377. 10.1016/j.chom.2011.09.002 22018237 PMC7172163

[B26] Hughes J. F. Coffin J. M. (2001). Evidence for genomic rearrangements mediated by human endogenous retroviruses during primate evolution. *Nat. Genet.* 29 487–489. 10.1038/ng775 11704760

[B27] Kapusta A. Kronenberg Z. Lynch V. J. Zhuo X. Ramsay L. a. Bourque G. (2013). Transposable elements are major contributors to the origin, diversification, and regulation of vertebrate long noncoding RNAs. *PLoS Genet.* 9:e1003470 10.1371/journal.pgen.1003470 PMC3636048 23637635

[B28] Katzourakis A. Gifford R. J. (2010). Endogenous viral elements in animal genomes. *PLoS Genet.* 6:e1001191 10.1371/journal.pgen.1001191 PMC2987831 21124940

[B29] Katzourakis A. Gifford R. J. Tristem M. Gilbert M. T. P. Pybus O. G. (2009). Macroevolution of complex retroviruses. *Science* 325:1512 10.1126/science.1174149 19762636

[B30] Keckesova Z. Ylinen L. M. J. Towers G. J. Gifford R. J. Katzourakis A. (2009). Identification of a RELIK orthologue in the European hare (*Lepus europaeus*) reveals a minimum age of 12 million years for the lagomorph lentiviruses. *Virology* 384 7–11. 10.1016/j.virol.2008.10.045 19070882 PMC3556577

[B31] Lavialle C. Cornelis G. Dupressoir A. Esnault C. Heidmann O. Vernochet C. (2013). Paleovirology of “syncytins”, retroviral env genes exapted for a role in placentation. *Philos. Trans. R. Soc. Lond. B Biol. Sci.* 368:20120507 10.1098/rstb.2012.0507 PMC3758191 23938756

[B32] Lepa A. Siwicki A. K. (2011). Retroviruses of wild and cultured fish. *Pol. J. Vet. Sci.* 14 703–709. 10.2478/v10181-011-0106-8 22439348

[B33] Liu H. Fu Y. Xie J. Cheng J. Ghabrial S. A. Li G. (2011). Widespread endogenization of densoviruses and parvoviruses in animal and human genomes. *J. Virol.* 85 9863–9876. 10.1128/JVI.00828—811 21795360 PMC3196449

[B34] Llorens C. Muñoz-Pomer A. Bernad L. Botella H. Moya A. (2009). Network dynamics of eukaryotic LTR retroelements beyond phylogenetic trees. *Biol. Direct* 4:41 10.1186/1745-6150-4-41 PMC2774666 19883502

[B35] Malik H. S. Henikoff S. Eickbush T. H. (2000). Poised for contagion: evolutionary origins of the infectious abilities of invertebrate retroviruses. *Genome Res.* 10 1307–1318. 10.1101/gr.145000 10984449

[B36] Marín I. (2010). GIN transposons: genetic elements linking retrotransposons and genes. *Mol. Biol. Evol.* 27 1903–1911. 10.1093/molbev/msq072 20228153

[B37] Micale L. Loviglio M. N. Manzoni M. Fusco C. Augello B. Migliavacca E. (2012). A fish-specific transposable element shapes the repertoire of p53 target genes in zebrafish. *PLoS ONE* 7:e46642 10.1371/journal.pone.0046642 PMC3485254 23118857

[B38] Nair S. Rein A. (2014). Antiretroviral restriction factors in mice. *Virus Res.* 193 130–134. 10.1016/j.virusres.2014.07.002 25018022 PMC4254267

[B39] Naville M. Chalopin D. Volff J.-N. (2014). Interspecies insertion polymorphism analysis reveals recent activity of transposable elements in extant coelacanths. *PLoS ONE* 9:e114382 10.1371/journal.pone.0114382 PMC4255032 25470617

[B40] Naville M. Warren I. A. Haftek-Terreau Z. Chalopin D. Brunet F. Levin P. (2016). Not so bad after all: retroviruses and long terminal repeat retrotransposons as a source of new genes in vertebrates. *Clin. Microbiol. Infect.* 22 312–323. 10.1016/j.cmi.2016.02.001 26899828

[B41] Patel M. R. Emerman M. Malik H. S. (2011). Paleovirology – ghosts and gifts of viruses past. *Curr. Opin. Virol.* 1 304–309. 10.1016/j.coviro.2011.06.007 22003379 PMC3190193

[B42] Paul T. A. Quackenbush S. L. Sutton C. Casey R. N. Bowser P. R. Casey J. W. (2006). Identification and characterization of an exogenous retrovirus from atlantic salmon swim bladder sarcomas. *J. Virol.* 80 2941–2948. 10.1128/JVI.80.6.29412948.2006. 16501103 PMC1395439

[B43] Qin S. Jin P. Zhou X. Chen L. Ma F. (2015). The role of transposable elements in the origin and evolution of micrornas in human. *PLoS ONE* 10:e0131365 10.1371/journal.pone.0131365 PMC4482582 26115450

[B44] Schartl M. Walter R. B. Shen Y. Garcia T. Catchen J. Amores A. (2013). The genome of the platyfish, *Xiphophorus maculatus*, provides insights into evolutionary adaptation and several complex traits. *Nat. Genet.* 45 567–572. 10.1038/ng.2604 23542700 PMC3677569

[B45] Shen C.-H. Steiner L. A. (2004). Genome structure and thymic expression of an endogenous retrovirus in zebrafish. *J. Virol.* 78 899–911. 10.1128/JVI.78.2.899-911.2004 14694121 PMC368747

[B46] Shi J. Zhang H. Gong R. Xiao G. (2015). Characterization of the fusion core in zebrafish endogenous retroviral envelope protein. *Biochem. Biophys. Res. Commun.* 460 633–638. 10.1016/j.bbrc.2015.03.081 25804638 PMC7092836

[B47] Varela M. Spencer T. E. Palmarini M. Arnaud F. (2009). Friendly viruses: the special relationship between endogenous retroviruses and their host. *Ann. N. Y. Acad. Sci.* 1178 157–172. 10.1111/j.1749-6632.2009.05002.x 19845636 PMC4199234

[B48] Volff J.-N. (2005). Genome evolution and biodiversity in teleost fish. *Heredity* 94 280–294. 10.1038/sj.hdy.6800635 15674378

[B49] Volff J.-N. (2006). Turning junk into gold: domestication of transposable elements and the creation of new genes in eukaryotes. *BioEssays* 28 913–922. 10.1002/bies.20452 16937363

[B50] Volff J.-N. Lehrach H. Reinhardt R. Chourrout D. (2004). Rretroelement dynamics and a novel type of chordate retrovirus-like element in the miniature genome of the tunicate *Oikopleura dioica*. *Mol. Biol. Evol.* 21 2022–2033. 10.1093/molbev/msh207 15254255

[B51] Walker R. (1969). Virus associated with epidermal hyperplasia in fish. *Natl. Cancer Inst. Monogr.* 31 195–207. 5393702

[B52] Warren I. A. Naville M. Chalopin D. Levin P. Berger C. S. Galiana D. (2015). Evolutionary impact of transposable elements on genomic diversity and lineage-specific innovation in vertebrates. *Chromosome Res.* 23 505–531. 10.1007/s10577-015-9493-9495 26395902

[B53] Wildschutte J. H. Williams Z. H. Montesion M. Subramanian R. P. Kidd J. M. Coffin J. M. (2016). Discovery of unfixed endogenous retrovirus insertions in diverse human populations. *Proc. Natl. Acad. Sci. U.S.A.* 113 E2326–E2334. 10.1073/pnas.1602336113 27001843 PMC4843416

